# Comparison between ultra-performance liquid chromatography with tandem mass spectrometry and a chemiluminescence immunoassay in the determination of cyclosporin A and tacrolimus levels in whole blood

**DOI:** 10.3892/etm.2013.1325

**Published:** 2013-10-04

**Authors:** YONG-WHA LEE

**Affiliations:** Department of Laboratory Medicine and Genetics, Soonchunhyang University Bucheon Hospital and Soonchunhyang University College of Medicine, Bucheon, Gyeonggi 420-767, Republic of Korea

**Keywords:** cyclosporin A, tacrolimus, ultra-performance liquid chromatography, tandem mass spectrometry, chemiluminescence immunoassay

## Abstract

Regular immunosuppressant drug monitoring is important for maintaining the drug concentrations of organ recipients within the therapeutic range. The standardized liquid chromatography-tandem mass spectrometry (LC-TMS) technique has been used for the accurate analysis of immunosuppressive drugs. In the present study, the performance of the recently developed high-throughput, rapid ultra-performance liquid chromatography combined with tandem mass spectrometry (UPLC-TMS) method was validated for the simultaneous measurement of cyclosporin A and tacrolimus in whole blood. The method of measuring cyclosporin A and tacrolimus using UPLC-TMS was established and the precision, limit of detection (LOD), limit of quantitation (LOQ) and matrix effect were validated. In addition, the performance of UPLC-TMS was compared with that of a chemiluminescence immunoassay (CLIA) in >3,400 clinical specimens. The UPLC-TMS revealed a within-run and between-run precision of <8% and showed a bias of <5%. The LOD and LOQ were 2.0 and 2.5 ng/ml for cyclosporin A, and 0.3 and 0.4 ng/ml for tacrolimus, respectively. Interference from the matrix was not observed. The CLIA measurements of cyclosporin A and tacrolimus showed correlations corresponding with the formulae: Concentration_(CLIA)_ = 1.18 × UPLC-TMS – 5.85; [95% CI: proportional, 1.16–1.19; constant, −6.86–(−4.81)] and Concentration_(CLIA)_ = 1.14 × UPLC-TMS – 0.38; [(95% CI: proportional, 1.13–1.14; constant, −0.35–(−0.43)], respectively. The majority of results were higher for the immunoassay than for the UPLC-TMS. The newly developed rapid UPLC-TMS method was suitable for use with a large therapeutic concentration range of the analyzed immunosuppressive drugs. Sample preparation was simple and it was possible to detect several immunosuppressants simultaneously, thus significantly lowering the cost of analysis. In conclusion, this method may contribute to improved accuracy and may be preferred to immunoassays for the routine clinical measurement of immunosuppressive drug concentrations in whole blood.

## Introduction

Numerous immunosuppressive drugs, including cyclosporin A, tacrolimus (previously termed FK 506), sirolimus (rapamycin), everolimus and mycophenolic acid, are used to prevent the rejection of transplanted organs or tissues (1). Narrow therapeutic indices, variable inter- and intra-individual pharmacokinetics and pharmacodynamics complicate drug dosing. To maintain drug concentrations in the therapeutic range and minimize their toxicity or the risk of organ rejection, regular immunosuppressant drug monitoring is required (2,3).

Several common and easily automated immunoassays are currently used to determine the concentrations of immunosuppressive drugs. These include: the enzyme multiplied immunoassay, radioimmunoassay, enzyme-linked immunosorbent assay, cloned enzyme donor immunoassay, chemiluminescence immunoassay (CLIA) and fluorescence polarization immunoassay (4,5). A primary disadvantage of these methods is that cross-reactions between drugs and metabolites may result in overestimation of the drug concentration with unacceptable bias. Furthermore, they are high in cost and are not able to assay multiple drugs simultaneously.

Liquid chromatography-mass spectrometry/mass spectrometry (LC-MS/MS) has the potential to circumvent the problems of poor specificity by separating drugs from metabolites and, therefore, is one of the most selective methods applied to therapeutic drug monitoring (TDM) (6). Several LC techniques using ultraviolet detection, mass spectrometry or tandem mass spectrometry (TMS) have been developed for the measurement of immunosuppressant drug concentrations (7–12).

In the present study, the performance of a recently developed high-throughput, rapid, ultra-performance liquid chromatography (UPLC) TMS method was analyzed, using common sample pretreatments for the simultaneous quantification of cyclosporin A and tacrolimus in whole blood. The analytical procedure was validated by the comparison of the UPLC-TMS results with those of CLIA for >3,000 clinical samples from transplant patients.

## Materials and methods

### Collection of specimens

Whole blood samples (stored in EDTA tube at 4ºC until tested) were collected from multiple transplant patients receiving cyclosporin A or tacrolimus during hospitalization or outpatient treatment between May 2011 and May 2013. All samples were anonymized and measured by two analytical methods (UPLC-TMS and CLIA) within three days of collection. The study was approved by the Institutional Review Board of Soonchunhyang University Bucheon Hospital (Buchneon, Korea). Informed consent was obtained from the patients

### UPLC-TMS

#### Materials and reagents

All solvents were LC-MS grade. Methanol and acetonitrile were obtained from Duksan Pure Chemicals (Gyeonggi, Korea) and formic acid, ammonium acetate and zinc sulfate heptahydrate were purchased from Sigma-Aldrich (St. Louis, MO, USA). The MassCheck^®^ immunosuppressants kit (Chromsystems Instruments and Chemicals GmbH, Munich, Germany) included six level calibrators and four level controls. Ascomycin served as an internal standard for tacrolimus and was obtained from Sigma-Aldrich, and cyclosporin D served as an internal standard for cyclosporin A and was obtained from United States Biological, Inc. (Salem, MA, USA).

#### Sample preparation

Calibrators and controls were reconstituted by dissolving in 2 ml LC-MS grade water for 10 min. The solutions were then agitated on a roller mixer for 2 h. The contents of the internal standard were reconstituted in 1 ml methanol. The volume of the internal standard was brought to 250 ml with acetonitrile.

Forty microliters of whole blood (obtained from calibrators, control materials and patient samples) were transferred into individual 1.5-ml tubes. Subsequently, 80 ml 0.1 M zinc sulfate solution was added to induce hemolysis. The samples were vortexed for 1 min, followed by the addition of 200 μl internal standard with acetonitrile to precipitate proteins. The contents were mixed until the samples were thoroughly dissolved and then centrifuged at 15,000 × g for 5 min. The supernatant was transferred to a V-bottomed microplate (Chromsystems Instruments and Chemicals GmbH) and sample aliquots were injected into the UPLC-TMS instrument for analysis.

#### UPLC-TMS measurements

UPLC was performed on a Waters Acquity^®^ UPLC system (Waters Corporation, Milford, MA, USA). The extract (20 μl) was injected via an autosampler into an Acquity UPLC C18 column (2.1×10 mm, 1.8 μm) and maintained at 55ºC in the column oven. LC separation was performed using a gradient profile of mobile phase A and B solutions, consisting of 2 mM ammonium acetate with 0.1% formic acid (v/v) in water and 2 mM ammonium acetate with 0.1% formic acid (v/v) in methanol, respectively. The flow rate was 400 μl/min and the running time was 1.8 min. The gradient program was 50% mobile phase B for 0.2 min, increased to 100% mobile phase B at 400 μl/min and followed by a change to 50% mobile phase B for 1 min. The total instrumental analysis time was 2.5 min, including re-equilibration of the column.

TMS was used to detect cyclosporin A, tacrolimus and their corresponding deuterium-labeled internal standards on a Waters Acquity^®^ TQ-Detector (Waters Corporation). At unit mass resolution, the mass analyzer had the following settings: cone voltage at 34 V; collision energy at 20 eV; source and desolvation temperatures of 130 and 350ºC, respectively; and desolvation gas flow at 800 l/h. The analysis was performed using electrospray positive ionization in the multiple reaction monitoring (MRM) mode: a mass to charge ratio (m/z) of cyclosporin A, tacrolimus, ascomycin and cyclosporin D of 1,219.9>1,202.5, 821.5>768.1, 809.5>756.1 and 1,234.0>1,216.6, respectively. Quantitation was performed using the TargetLynx Manager in the Waters MassLynx 4.1 software (Waters Corporation) by the linear regression of the peak area ratios of cyclosporin A/cyclosporin D and tacrolimus/ascomycin against the calibrator concentrations with 1/x weighting.

#### CLIA

The EDTA-whole blood sample was extracted with a protein precipitation reagent comprising methanol and zinc sulfate and then centrifuged at centrifuged at 15,000 × g for 4 min. The supernatant obtained was recovered for analysis using the Abbott Architect i2000 system (Abbott Diagnostics, Abbott Park, IL, USA). Cyclosporin A or tacrolimus in the specimen bound to microparticles in the Abbott reagent that were coated with mouse antibodies raised against these drugs. After a brief period, an acridinium-labeled drug conjugate was added to the reaction mixture. This compound competed with the drug in the patient specimen for the available binding sites on the microparticles. Following incubation, the microparticles were washed and trigger solutions were added to the reaction mixture. The resulting chemiluminescent signal was expressed as relative light units. Due to the competitive binding nature of this reaction, an indirect correlation was observed between the quantity of drug and the relative light units detected by the system optics.

### Method validation

#### Precision

The within- and between-run precision of the UPLC-TMS method was assessed using duplicate level 1–4 serum control material samples for two days.

#### Limit of quantification (LOQ) and limit of detection (LOD)

The LOQ was determined using pooled samples. Pooled specimens were diluted with the blank pool to generate three concentrations of cyclosporin A and tacrolimus. Each sample was measured five times per pool, including the lowest pool concentration. The LOQ was defined as the concentration corresponding to the 20% coefficient of variation (CV) and >10:1 signal to noise ratio. LOD was defined as the lowest concentration corresponding to a >3:1 signal to noise ratio.

#### Matrix effect

The matrix effect was evaluated by a continuous infusion of internal standard (IS) at a flow rate of 20 μl/min into the effluent from the column, prior to its introduction into the MS system. Ion suppression/enhancement was analyzed on blood and water matrices by injecting 2 μl of pretreated blood and water into the MS/MS system and recording the MRM signal of the IS.

#### Method comparison

The UPLC-TMS method was compared with the CLIA method in the analysis of 3,469 clinical specimens obtained from various transplant patients receiving cyclosporin A or tacrolimus during hospitalization or outpatient clinic visits. The samples were distributed evenly from low to high concentrations.

#### Statistical analysis

Descriptive statistical analysis of the data was accomplished using Analyse-it for Microsoft Excel (Analyse-it Software Ltd., Leeds, UK). P<0.05 was considered to indicate a statistically significant difference. Passing and Bablok regression and a Bland-Altman plot were performed for comparison.

## Results

### Chromatograms of cyclosporin A and tacrolimus using UPLC-TMS

**Figure 1:**
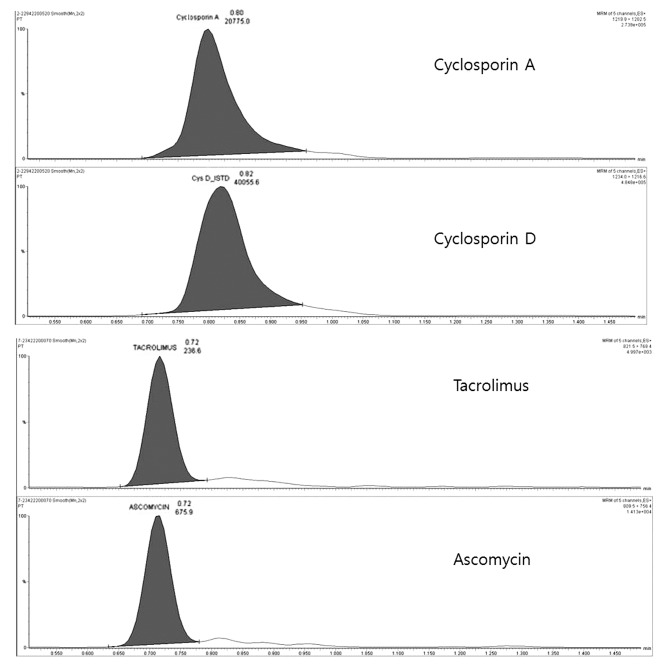
Chromatograms of cyclosporin A, cyclosporin D (internal standard of cyclosporin A), tacrolimus and ascomycin (internal standard of tacrolimus) in whole blood measured by ultra-performance liquid chromatography with tandem mass spectrometry.

Four isolated peaks were chromatographically separated, corresponding to cyclosporin A and tacrolimus with their internal standards, as shown in Fig. 1. The retention times of cyclosporin A and tacrolimus were 0.80 and 0.74 min, respectively. These are identical to the retention times of the calibration and internal standards (Fig. 1).

### Performance validation of UPLC-TMS

The assay precision performance is summarized in Table I, which shows within-run and between-run quality control precision. Overall, the CVs were less than the maximum CV tolerated and widely accepted for drug measurements (15%) and showed a bias of <5% (13).

The LOD and LOQ were 2.0 ng/ml (CV, 20.5) and 2.5 ng/ml (CV, 16.0%), respectively, for cyclosporin A. The LOD and LOQ were 0.3 ng/ml (CV, 21.1%) and 0.4 ng/ml (CV, 18.1%), respectively, for tacrolimus.

Interference from the matrix was not observed. The ion suppression tests showed that neither of the immunosuppressant drugs exhibited ion suppression at their elution times. Throughout the run, the sensitivity increased due to the increasing methanol concentration in the gradient.

**Figure 2:**
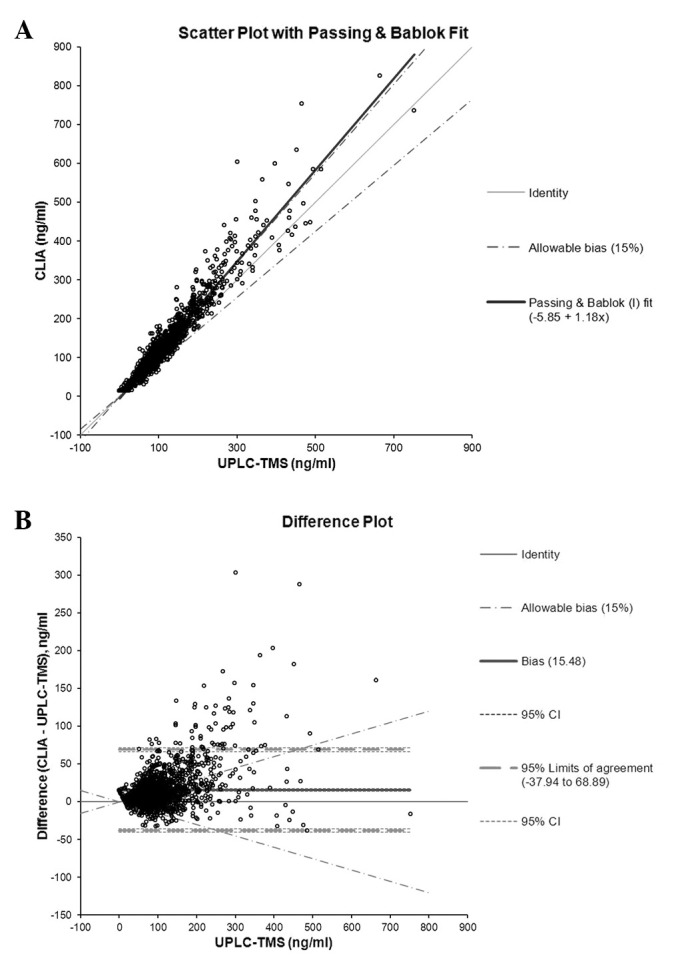
Comparison of whole blood cyclosporin A measured by ultra-performance liquid chromatography with tandem mass spectrometry and the chemiluminescence immunoassay method. (A) Passing and Bablok regression plot and (B) Bland-Altman plot.

**Figure 3:**
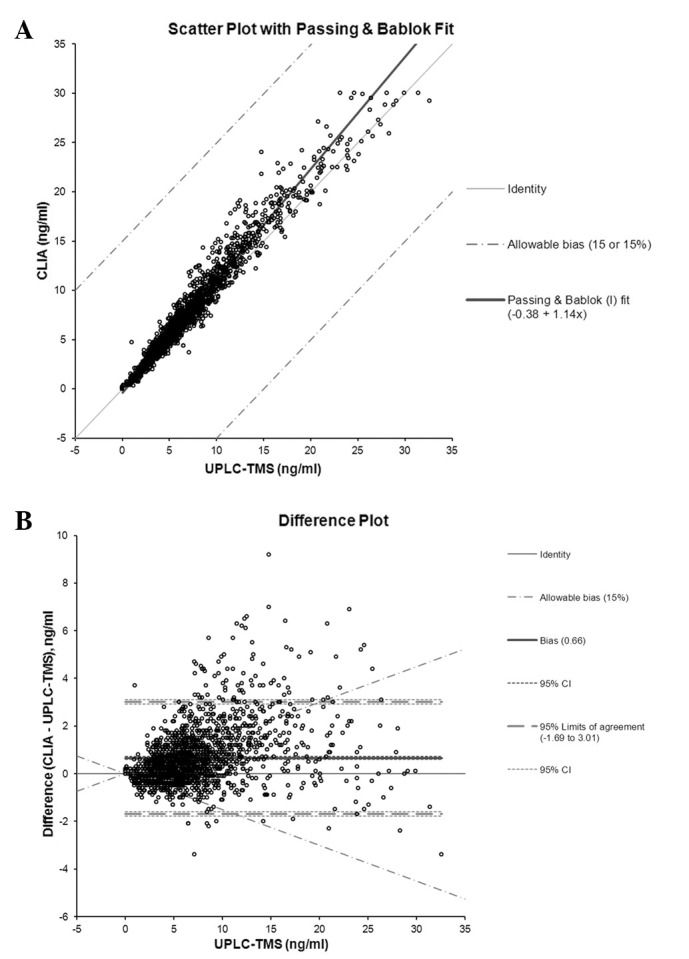
Comparison of whole blood tacrolimus measured by ultra-performance liquid chromatography with tandem mass spectrometry and the chemiluminescence immunoassay method. (A) Passing and Bablok regression plot and (B) Bland-Altman plot.

In the comparative study, the results of UPLC-TMS measurements were comparable to those of CLIA by Passing and Bablok regression analysis. The slope of Concentration_(CLIA)_ was 1.18, the intercept was −5.85 [(Concentration_(CLIA)_ = 1.18 × UPLC-TMS – 5.85; 95% CI: proportional, 1.16–1.19; constant, −6.86–(−4.81)] and the mean difference between the two methods was 15.5 ng/ml (95% CI: proportional, 14.1–16.8) for cyclosporin A, based on the Bland-Altman plot (Fig. 2). For tacrolimus, the slope of Concentration_(CLIA)_ was 1.14, the intercept was −0.38 [Concentration_(CLIA)_ = 1.14 × UPLC-TMS – 0.38, 95% CI: proportional, 1.13–1.14; constant, −0.35–(−0.43)] and the mean difference between the two methods was 0.66 ng/ml (95% CI 0.60 to 0.71) (Fig. 3). There was a systematic deviation in the blood levels measured by UPLC-TMS compared with those measured by CLIA for the two drugs. For cyclosporin A, the concentrations measured by UPLC-TMS were ~18% lower than those measured by CLIA. For tacrolimus, the concentrations measured by UPLC-TMS were ~14% lower. The majority of the results were higher for the immunoassay than for UPLC-TMS.

## Discussion

The use of spectrometry-based technology for routine quantitative immunosuppressant drug monitoring in clinical laboratories is increasing. In this study, evaluation of the newly developed high-throughput UPLC-TMS technique in the measurement of cyclosporin A and tacrolimus in clinical samples was performed. The method described in the present study was validated and shown to be selective, rapid and robust with little interference from compromising peaks.

Linearity was determined using 10 calibration curves for the immunosuppressant drugs in whole blood. The calibration concentrations covered the entire range of the expected patient sample concentrations. The calibration curves were linear for cyclosporin A, and tacrolimus was within the calibration range. The R^2^ coefficients for the calibration curves were >0.99 for the two drugs.

The LODs allowed the concentration of each analyte to be measured with accuracy and precision. Thus, the described methods exhibited sufficient sensitivity for diagnostic purposes. The 2007 European Consensus Conference on Tacrolimus Optimization recommended the use of tacrolimus assays with an LOQ of <1.0 ng/ml to support low dose tacrolimus therapy monitoring (14).

The within- and between-run precision analysis of the UPLC-TMS method showed that the values of cyclosporin A and tacrolimus obtained had a CV of <8.0% per drug, which is consistent with data reported by the manufacturer of the UPLC-TMS system.

A comparative analysis of >3,400 patient samples was conducted with CLIA. As expected, in the majority of the blood samples, the levels of cyclosporin A and tacrolimus were systematically higher when measured by CLIA, due to significant metabolite or structural analogue cross-reactivity; however, reading variability was also dependent upon sample collection time and individual metabolic characteristics. Therefore, these confounding factors contribute to the difficulty associated with a UPLC-TMS correlational study.

The concentrations measured by UPLC-TMS were observed to be ~18% lower than those measured by CLIA for cyclosporin A. The gradient is the primary reason for this deviation. In addition, similar results for cyclosporin were demonstrated in a previous study (15).

For tacrolimus, however, varying results from comparisons of UPLC-TMS and CLIA have been reported (13,16). Such variations may be in part due to differences in patient population. These factors suggest a requirement for more accurate drug measurement methods. Thus, the high selectivity of the UPLC-TMS method may prevent the overestimation of drug concentrations in patient samples.

In the present study, the newly developed UPLC-TMS method was shown to perform well for a wide range of therapeutic immunosuppressant drug concentrations. In addition, the sample preparation was simple and the method allowed the assay of multiple drugs simultaneously, while also being high-throughput. Thus, UPLC-TMS used in this capacity significantly lowers the cost of analysis. In conclusion, this method may improve the accuracy, speed and expense associated with the routine measurement of immunosuppressive drug concentrations in whole blood compared with other typical immunoassays.

## Figures and Tables

**Table I:** Within- and between-run precision for cyclosporin A and tacrolimus control materials.

		Within-run			Between-run		
			
Material	Target (ng/ml)	Mean (ng/ml)	SD	CV (%)	Mean (ng/ml)	SD	CV (%)
Cyclosporin A							
Level 1	53.0	53.2	2.1	3.9	53.6	2.4	4.4
Level 2	261.0	251.0	9.5	3.8	244.4	10.3	4.2
Level 3	495.0	485.9	25.4	5.2	479.0	29.6	6.2
Level 4	1140.0	1121.6	51.9	4.6	1105.6	62.4	5.6
Tacrolimus							
Level 1	2.8	2.8	0.11	3.9	2.8	0.14	5.0
Level 2	7.8	7.7	0.31	4.0	7.6	0.38	5.0
Level 3	15.5	15.4	0.70	4.5	15.5	0.81	5.2
Level 4	32.6	32.6	2.36	7.2	32.6	2.59	7.9

CV, coefficient of variation.
